# Supplementary material for: Correlation between two distant quasiparticles in separate superconducting islands mediated by a single spin
Source: Nat Commun. 2024 Apr 24;15:3465. doi: 10.1038/s41467-024-47694-7 (PMC11043349; doi:10.1038/s41467-024-47694-7)
Supplement: Supplementary file 1 — Supplementary Information [file 41467_2024_47694_MOESM1_ESM.pdf]

Correlation between two distant quasiparticles in separate superconducting islands mediated by a single spin: Supplementary Information

Juan Carlos Estrada Saldaña[1], Alexandros Vekris[1,2], Luka Pavešič[3,4], Rok Žitko[3,4,5], Kasper Grove-Rasmussen[1], and Jesper Nygård[1,6]

[1] *Center for Quantum Devices, Niels Bohr Institute, University of Copenhagen, 2100 Copenhagen, Denmark*

[2] *Sino-Danish College (SDC), University of Chinese Academy of Sciences*

[3] *Jožef Stefan Institute, Jamova 39, SI-1000 Ljubljana, Slovenia*

[4] *Faculty of Mathematics and Physics, University of Ljubljana, Jadranska 19, SI-1000 Ljubljana, Slovenia*

[5] *e-mail: rok.zitko@ijs.si and*

[6] *e-mail: nygard@nbi.ku.dk*

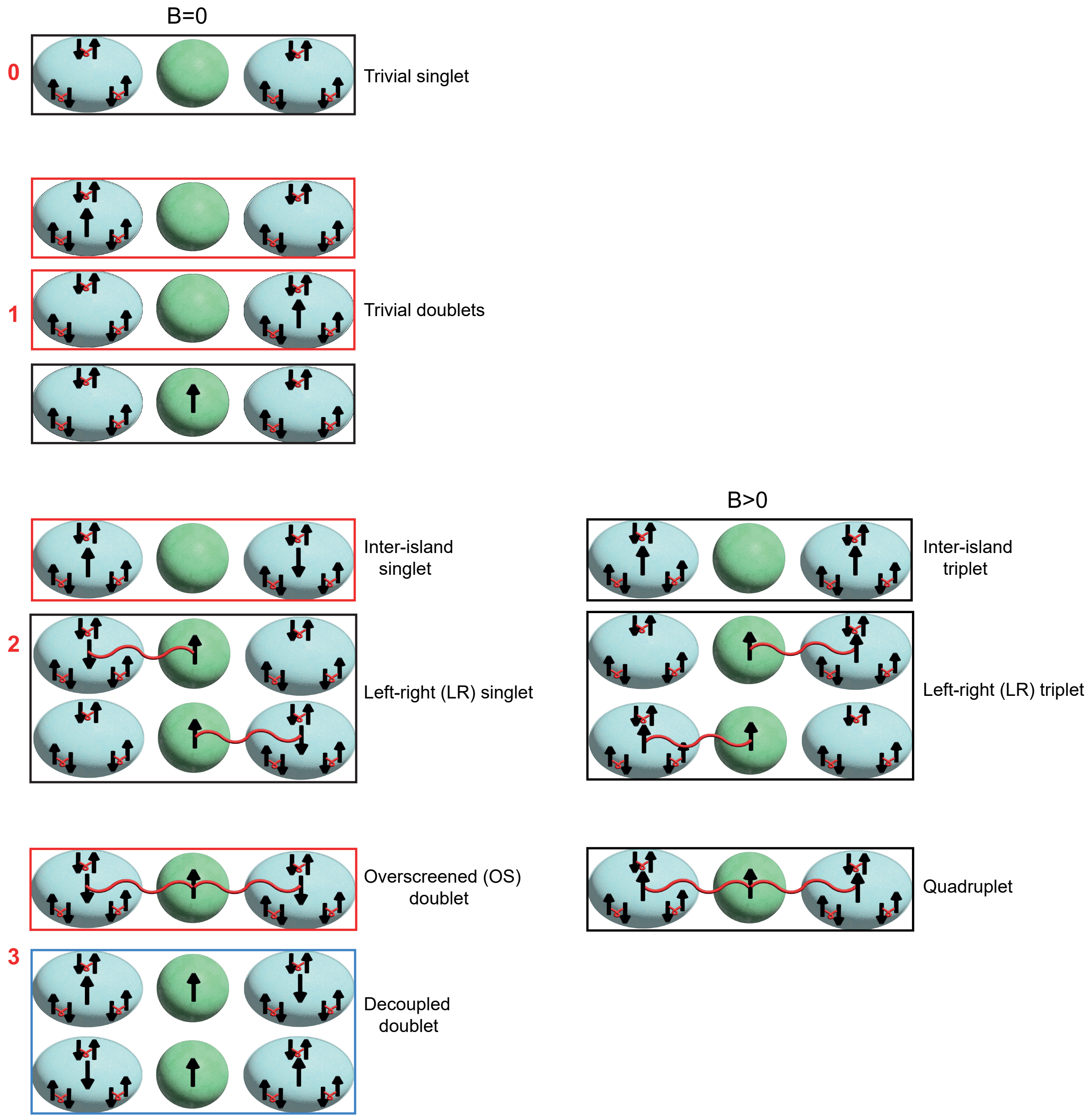

Supplementary Figure S 1. **Ground state for different number of local moments.** Depictions of the wavefunction of the ground state of the SI-QD-SI chain for different total number of LMs. Red frames indicate that the state is the GS only for $E_c > \Delta$, while blue frames indicate that the state is only the GS for $E_c < \Delta$. States with black frames can be the GS at any finite E_c. States in the left column can be the GS at $B = 0$, and those in the right column may become the GS only at $B > 0$. The states in the left column may become the GS at $B > 0$ independently of the E_c/Δ ratio. The squiggles indicate some degree of entanglement between the spins (partial or full).

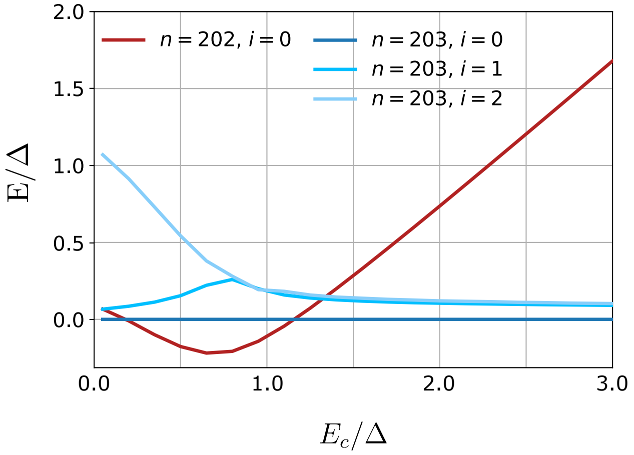

Supplementary Figure S 2. **Spectra vs. charging energy.** Calculated energies of the singlet ground state (red) and the first three doublet excitations (blue) vs. charging energy E_c. The energies are given relative to the ground state in the doublet sector. Here $U/\Delta = 60$, $\Gamma/U = 0.08$ The calculation of excitation energies ($i > 0$) for $E_c/\Delta < \frac{1}{4}$ is not reliable, see Sec. II D of L. Pavesic and R. Zitko, Phys. Rev. **B** 95, 085121 (2017).

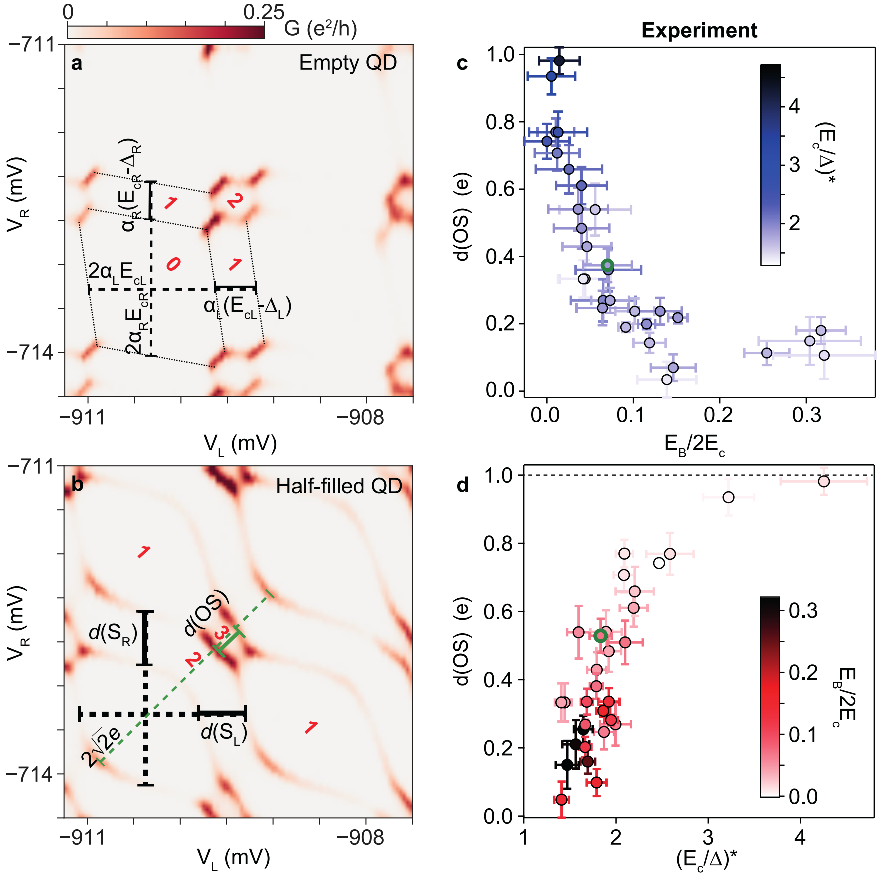

Supplementary Figure S 3. **Binding of two quasiparticles to one spin.** **a,b** Stability diagrams presented as zero-bias G versus V_{L} and V_{R} for (a) empty QD, (b) QD filled with one LM. The total number of LMs in the device is indicated in red numbers. Solid bars in (a) normalized by the dashed bars provide estimates for the E_{c}/Δ ratios independent of the gate-to-energy conversion factors, denoted as $(E_{\mathrm{c}}/\Delta)^{*}$. Solid horizontal and vertical bars in (b) minus those in (a), normalized by the dashed bars, provide $E_{\mathrm{B}}/2E_{\mathrm{c}}$. The diagonal solid bar of length d(OS) in (b) measures the size of the OS doublet when it is the ground state. The diagonal dashed bar measures the gate voltage needed to add two electrons in each SI, and we use it as a normalization factor for the size of the OS region. **c** Normalized OS region size in 26 QD shells, $2\sqrt{2}d(\mathrm{OS})$, against $E_{\mathrm{B}}/2E_{\mathrm{c}}$. $(E_{\mathrm{c}}/\Delta)^{*} = 1.3 - 4.2$, given by the color scale. Left-right differences in E_{B} and E_{c}/Δ are 0-30%. $U/\Delta = 2 - 4$ measured from Coulomb diamonds spectroscopy. **d** Same data as in (c), plotted against $(E_{\mathrm{c}}/\Delta)^{*}$. $E_{\mathrm{B}}/2E_{\mathrm{c}} = 0 - 0.32$, given by the color scale. The green circle indicates the data point extracted from (a, b). Error bars are based on the full width at half-maximum of the conductance lines used in the extraction of the data. The assumption that the size of d(OS) is a good measure of excitation energy, and its relation to other model parameters is gauged with numerical calculations in Supplementary Fig. S6.

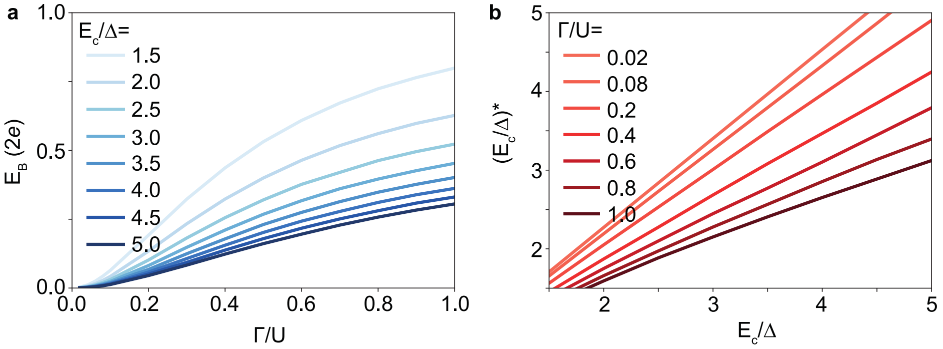

Supplementary Figure S 4. **Relation between parameters extracted from stability diagrams and model parameters.** **a**,**b** Calculations using the DMRG of the relations between (a) the binding energy E_B and the hybridisation strength Γ and (b) the effective ratio $(E_\mathrm{c}/\Delta)^*$ (extracted from the stability diagram, as explained in the Supplementary Fig. S3) and the ratio E_c/Δ (raw model parameters). These results establish a one-to-one relation between theory parameters (Γ, E_c, U), and experimentally accessible values (E_B, $(E_c/U)^*$) which quantify the coupling strength and the Coulomb repulsion.

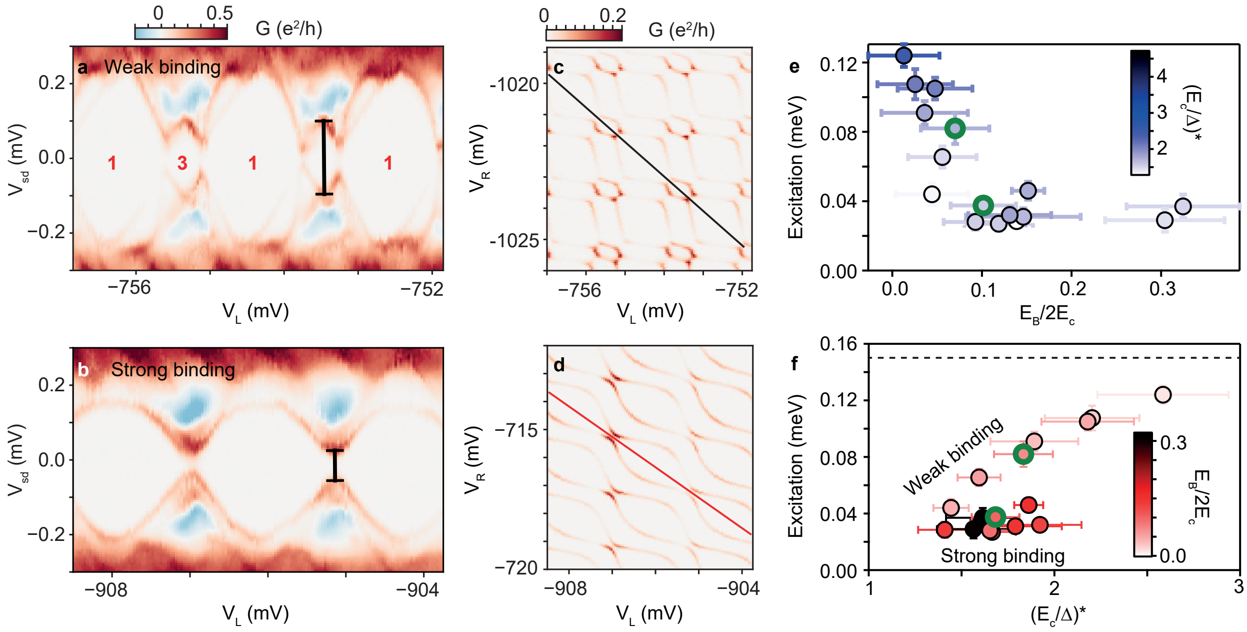

Supplementary Figure S 5. **Overscreened doublet to left-right singlet excitations. a,b** Examples of bias spectra for **(a)** weak and **(b)** strong E_B. For weak binding, the transition between the 3 LM and 1 LM doublets are well resolved, while for strong binding there is mixing between the OS and decoupled doublets producing smooth cross-overs. **c,d** Zero-bias G stability diagrams with 1 LM fixed in the QD. Solid lines indicate the V_L, V_R trajectories in **(a)**,**(b)**. In these trajectories, the number of LMs in the SIs is varied between 2 and 0 in alternation. Red numbers **(a)**,**(b)** indicate the total number of LMs in the GS. **e**, **f** Compilation of doublet→singlet excitation energies in the middle of the OS GS sector for 16 QD shells versus **(e)** $E_\mathrm{B}/2E_\mathrm{c}$ and **(f)** $(E_\mathrm{c}/\Delta)^*$. Left-right differences in $E_\mathrm{B}/2E_\mathrm{c}$ and $(E_\mathrm{c}/\Delta)^*$ are less than 30% and 25%, respectively. To avoid zero-bias offset issues, the excitation energies are measured as the average of the sum of the addition and removal energies. The sum is given by vertical bars in the examples in **(a)**, **(b)**. Green circles are the excitation energy values extracted from these examples. Error bars are based on the full width at half-maximum of the conductance lines used in the extraction of the data. In **(f)**, the weak-binding data is expected to fully saturate at an excitation energy equal to $U/2 = 0.15$ meV (indicated by a dashed line), where U is measured from Coulomb-diamonds spectroscopy.

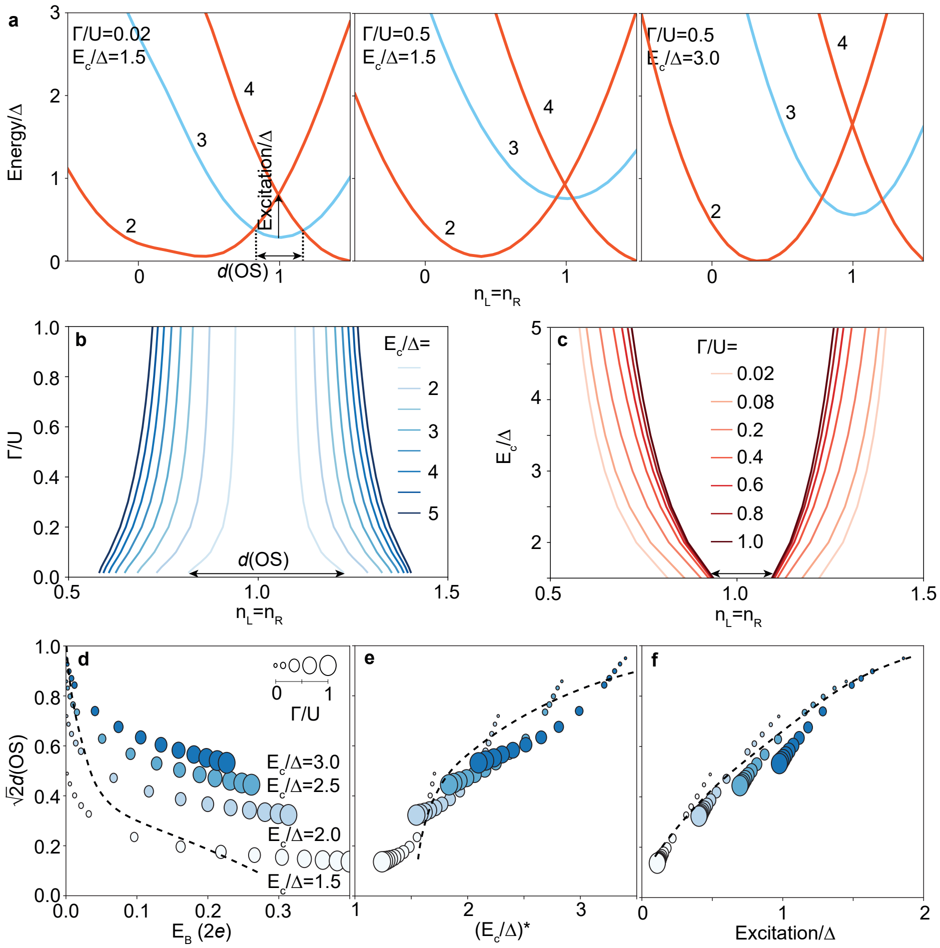

Supplementary Figure S 6. **Calculations of the overscreened doublet size and comparison to parameters. a** Calculated charge parabolas versus rigid shift of the gate-induced charge in the two SIs $n_{\mathrm{L}} = n_{\mathrm{R}}$, for fixed gate-induced charge in the QD $\nu = 1$. The sweeps correspond to the direction of the green diagonal dashed line in Supplementary Fig. S3b. The parabolas are tagged by the total charge in the system referenced to an even integer number of electrons in each of the SIs. The size of the overscreened doublet, $d(\mathrm{OS})$, and the doublet to singlet excitation energy are indicated by horizontal and vertical arrows. An increase in Γ/U or a reduction in E_{c}/Δ reduce the excitation energy and $d(\mathrm{OS})$. **b**, **c** Parabola crossings versus (b) Γ/U for different E_{c}/Δ and versus (c) E_{c}/Δ for different Γ/U. $d(\mathrm{OS})$ corresponds to the distance between the curves, indicated by horizontal arrows in the innermost pair of curves. **d**, **e**, **f** Calculated $\sqrt{2}d(\mathrm{OS})$ versus (d) E_{B}, (e) $(E_{\mathrm{c}}/\Delta)^{*}$, and (f) excitation energy normalized by Δ. The parameter region encompassed by the experiment corresponds approximately to the left of the dashed lines. $\sqrt{2}d(\mathrm{OS})$ grows approximately linearly with the excitation energy, showing that the former is an appropriate measure of the latter and enabling the comparisons made in Supplementary Fig. S3 between these two quantities.

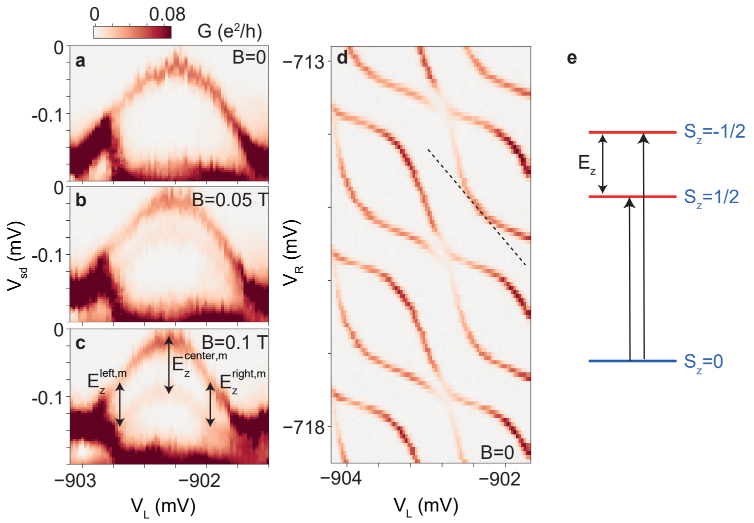

Supplementary Figure S 7. **Zeeman splitting of left-right singlet to doublet excitations.** **a-c** Magnetic field, B, dependence of the G versus source-drain bias, $V_{\rm sd}$, with the gates $V_{\rm L}$ and $V_{\rm R}$ swept along the dashed line in the zero-bias G stability diagram in (**d**). For simplicity, only $V_{\rm L}$ is indicated. The QD is half-filled, left-right binding energies are approximately symmetric and strong ($E_{\rm B}/2E_{\rm c} = 0.15 > \mathcal{E}$), and $E_{\rm cL}/\Delta_{\rm L} \approx E_{\rm cR}/\Delta_{\rm R} = 1.9$. The curved line in the measurement represents singlet→doublet excitations with gate-dependent state mixing, with the doublet state splitting into spin-up and spin-down components as B is increased. In (**c**), Zeeman energies at three spots in the split curve are indicated by double-headed arrows; they highlight that the splitting is larger at the center of the curve. The color scale is saturated to highlight the faint excitations. The g factors of the three device components are fairly symmetric: $g_{\rm L} = 12.6$, $g_{\rm N} = 15$ and $g_{\rm R} = 11$. The Zeeman splitting at the center is $E_{\rm z}^{\rm center,m} = 85 \pm 18$ μeV, which matches well the Zeeman splitting of the trivial doublet excitation $E_{\rm z}^{\rm center} = g_{\rm N}\mu_{\rm B}B = 87$ μeV as expected. At the left and right sides, the Zeeman splitting is $E_{\rm z}^{\rm left,m} = E_{\rm z}^{\rm right,m} = 67 \pm 16$ μeV. The expected Zeeman splittings three-quarters of the way (in gate voltage) to the OS doublet are $E_{\rm z}^{\rm left} = E_{\rm z}^{\rm right} = 0.5g_{\rm N}\mu_{\rm B}B + 0.5(n_{\rm L}g_{\rm L} + n_{\rm R}g_{\rm R})\mu_{\rm B}B$, where $n_{\rm L}$ and $n_{\rm R}$ are the gate-induced charges in the left and right SIs. These evaluate as $E_{\rm z}^{\rm left} = 54$ μeV for $n_{\rm L} = 0.75$, $n_{\rm R} = 0.25$, and $E_{\rm z}^{\rm right} = 68$ μeV for $n_{\rm L} = 0.25$, $n_{\rm R} = 0.75$, matching within error bars the measurements. **e** Schematics of singlet→doublet excitations (arrows). The energy difference between the excitations provides a measure of the Zeeman energy.

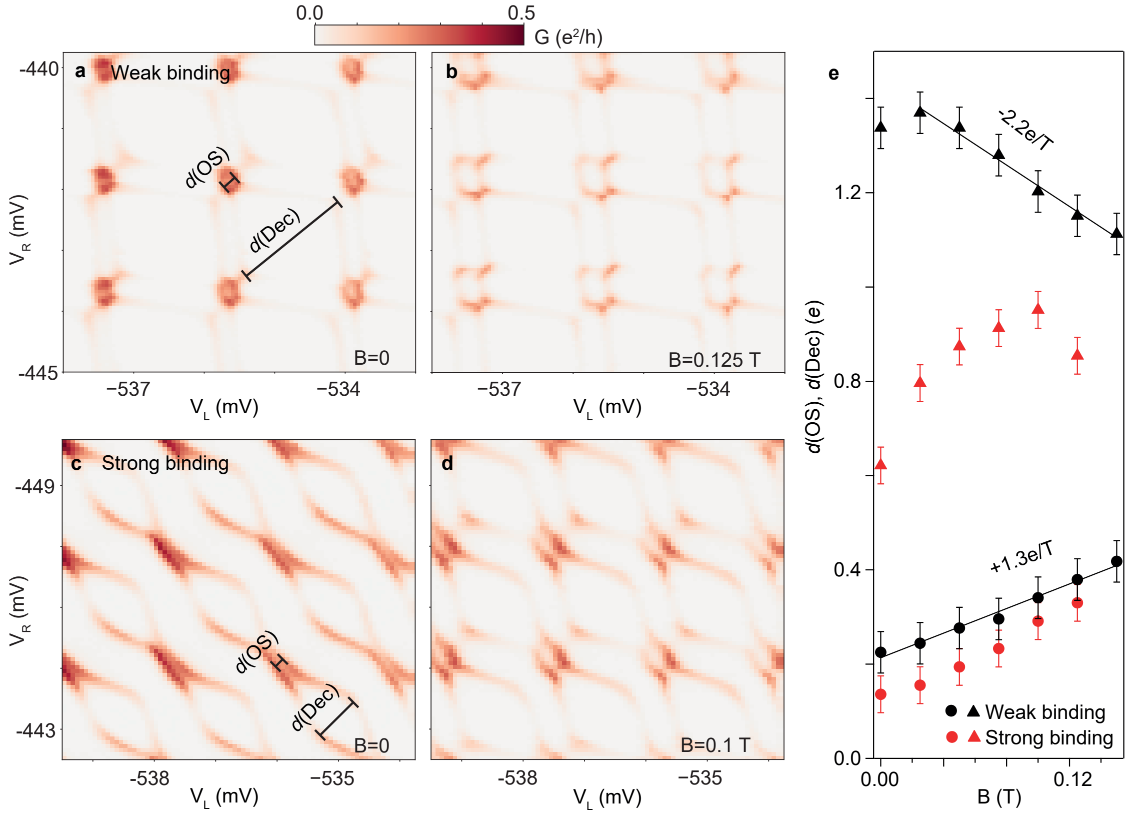

Supplementary Figure S 8. **Evolution of the stability diagram in a magnetic field. a-d** Zero-bias conductance stability diagrams recorded at different B indicated on each plot. The device parameters are identical to those of Fig. 4, i.e. (**a,b**) $E_\mathrm{B}/2E_\mathrm{c} = 0.04$ (weak binding), $E_\mathrm{c}/\Delta = 1.45$ and (**c,d**) $E_\mathrm{B}/2E_\mathrm{c} = 0.32$ (strong binding), $E_\mathrm{c}/\Delta = 1.65$. **e** B dependence of the diagonal sizes in the 3 LM (circles) and 1 LM (triangles) sectors extracted from stability diagrams for weak (black symbols) and strong binding (red symbols). The sizes at $B = 0$ are given by the bars in (**a**), (**b**). Lines are fits to the weak-binding data, with slopes indicated above each line. The data is qualitatively consistent with the Zeeman shifts of the excitation energies shown in Fig. 4.

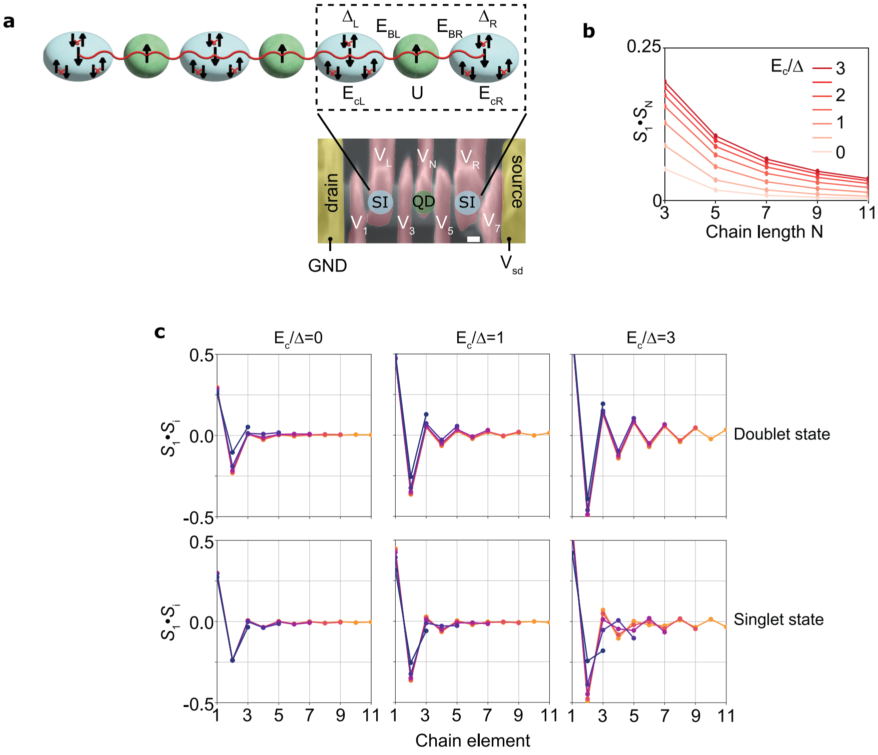

Supplementary Figure S 9. **Chain of Bogoliubov quasiparticles entangled by electron spins.** **a** Alternating chain of superconducting islands (SI) and quantum dots (QD). Each SI carries one Coulomb-blockaded quasiparticle, each QD one Coulomb-blockaded spin. In this arrangement, the Yu-Shiba-Rusinov interaction antiferromagnetically binds neighbouring local moments (LMs) within the chain. For equal bindings each QD spin is overscreened by two adjacent quasiparticles. Our device represents a minimal building block of this chain. **b** Calculated correlations $\langle \mathbf{S}_i \cdot \mathbf{S}_j \rangle$ between the spins at the ends of the chain versus chain length for a range of Coulomb repulsion E_c. See Methods for details. **c** Correlations between the first spin, S_1, and the spin in position i, S_i, for $i = 1, \ldots, L$, where L is the chain length. Each panel contains the results for chains of different odd lengths (colours), $L = 3, 5, 7, 9$ and 11. Left, central and right panels correspond to $E_\mathrm{c}/\Delta = 0$, 1 and 3. Top panels examine the correlations in the doublet ground state, while bottom ones examine correlations in the singlet ground state. In the doublet state, the correlations cross zero between adjacent elements (quantum dots or superconducting islands) for $E_\mathrm{c}/\Delta = 1$ and 3, indicating quasi-long-range order, typical of one-dimensional magnetic systems. The quasi-long-range order is lost for $E_\mathrm{c}/\Delta = 0$ and for any E_c/Δ value in the singlet state. Other parameters are $U/\Delta = 5$ and $\Gamma/U = 0.4$. This figure emphasizes the high degree of self-similarity of spin correlations in the doublet state, demonstrated by the good degree of overlap of the results for different L.
